# Why Do Forward Maskers Affect Auditory Intensity Discrimination? Evidence from "Molecular Psychophysics"

**DOI:** 10.1371/journal.pone.0099745

**Published:** 2014-06-17

**Authors:** Daniel Oberfeld, Patricia Stahn, Martha Kuta

**Affiliations:** Section Experimental Psychology, Department of Psychology, Johannes Gutenberg-Universität Mainz, Mainz, Germany; University of Leicester, United Kingdom

## Abstract

Nonsimultaneous maskers can strongly impair performance in an auditory intensity discrimination task. Using methods of molecular psychophysics, we quantified the extent to which (1) a masker-induced impairment of the representation of target intensity (i.e., increase in internal noise) and (2) a systematic influence of the masker intensities on the decision variable contribute to these effects. In a two-interval intensity discrimination procedure, targets were presented in quiet, and combined with forward maskers. The lateralization of the maskers relative to the targets was varied via the interaural time difference. Intensity difference limens (DLs) were strongly elevated under forward masking but less with contralateral than with ipsilateral maskers. For most listeners and conditions, perceptual weights measuring the relation between the target and masker levels and the response in the intensity discrimination task were positive and significant. Higher perceptual weights assigned to the maskers corresponded to stronger elevations of the intensity DL. The maskers caused only a weak increase in internal noise, unrelated to target level and masker lateralization. The results indicate that the effects of forward masking on intensity discrimination are determined by an inclusion of the masker intensities in the decision variable, compatible with the hypothesis that the impairment in performance is to a large part caused by difficulties in directing selective attention to the targets. The effects of masker lateralization are evidence for top-down influences, and the observed positive signs of the masker weights suggest that the relevant mechanisms are located at higher processing stages rather than in the auditory periphery.

## Introduction

The intensity of an auditory event is one of the basic stimulus attributes, and judgments of auditory intensity are important for behavior. For instance, the intensity change of an approaching sound provides information regarding when this object would reach the observer (time-to-contact [1]).

Non-simultaneous maskers temporally separated from a target sound by less than 500 ms can have a dramatic impact on performance in an auditory intensity discrimination task, with the intensity difference limen (DL) being up to 20 dB higher than in quiet (e.g., [2], [3], [4], [5], [6]). An important finding is that the DL elevation (i.e., the difference between the intensity DL under masking and the DL in quiet) caused by an intense masker (e.g., 90 dB SPL) is higher for midlevel standards (e.g., 60 dB SPL) than for standards with a low (30 dB SPL) or high (90 dB SPL) sound pressure level [7]. This so-called *mid-level hump* [7] places constraints on explanations of the effects of non-simultaneous masking (for a discussion see [3]). In addition, the finding that backward maskers presented after the target cause the same effect as forward maskers [2], [4], [8] is difficult to explain by processes in the auditory periphery and suggests an involvement of more central mechanisms [9].

Several explanations for the effects of non-simultaneous masking have been proposed (an in-depth discussion can be found in [3]). Zeng et al. [7] suggested that the relatively slow recovery of low spontaneous-rate neurons in the auditory nerve [10] creates a "coding gap" for midlevel standards if an intense forward masker is presented. The *referential encoding hypothesis* [8], [11] attributes the effects of non-simultaneous maskers to the use of a less precise type of memory representation than in a condition without masker (cf. [12]). Carlyon and Beveridge [11] proposed that the masker-induced DL elevations are due to variability in the loudness representation of the target, induced by systematic changes in target loudness caused by the masker (cf. [13]). Although these three explanations are rather different from each other, a common assumption is that the maskers degrade the representation of target intensity, either already at the level of the auditory nerve or at later processing stages. Recent data from our lab suggested a different hypothesis concerning the origin of the masker-induced reduction in intensity discrimination performance. The maskers might not affect the representation of target intensity in the sense of increased internal noise [14] effective for the targets, that is, a higher variability of the target representations. Instead, the impairment in performance might be due to the decision being influenced by the representations of masker intensity, although the maskers convey no information concerning the intensity of the targets and the masker-related intensity information should therefore be ignored. In two experiments [4], [5] we found that the effects of the maskers were increased in conditions where the maskers and targets were grouped together (i.e., a masker and the following target were perceived as one unitary object), relative to conditions favoring the processing of the maskers and the targets as two separate auditory objects [15]. These effects could be explained by object-based attention [16]. According to this important concept from cognitive psychology, it is more difficult to selectively attend to a feature within an object than to attend to one object while ignoring another object (e.g., [16], [17]). Thus, our results [4], [5] suggest that the masker-induced impairment in performance might be caused by difficulties in directing selective attention to the targets while ignoring the task-irrelevant maskers [4], [5].

In the present study, we propose a simple observer model for intensity discrimination under masking, based on a signal-detection theory framework, that does not require strong assumptions concerning the exact physiological mechanisms (e.g., peripheral versus central). The model shows that on a general level there are only three potential effects maskers can have on performance in an auditory intensity discrimination task. We then discuss how behavioral data collected in a "molecular psychophysics" [18] approach can be used to test which of these effects are effective in intensity discrimination under forward masking. In turn, the different proposed mechanisms introduced above are discussed in relation to the patterns of results, and we demonstrate that the results provide information concerning the potential physiological origins of the effects.

In an intensity discrimination task, the maskers could exert three different effects:

Effect (A). The maskers might alter the mean value of the representations of target intensity in the auditory system. For example, a forward masker could cause response suppression in auditory nerve neurons (e.g., [19]), resulting in a lower spike count produced in response to the target, relative to a situation where the target is presented in quiet. At higher processing stages (e.g., primary auditory cortex), enhancement rather than suppression of the neural response by a preceding sound is sometimes observed [20], although as will be discussed below it is unlikely that the stimulus configuration used in typical experiments on intensity discrimination under nonsimultaneous masking would lead to response enhancement. On the perceptual level, however, an intense sound like a forward masker can result in increased loudness of a temporally proximal target sound ("loudness enhancement"; [13], [21]).

Effect (B). The maskers might increase the variance of the representations of target intensity, which is typically modeled as an increase in *internal noise* [14]. Such an effect might for example be caused by suppression of the neural response to the target, which can result in a lower signal-to-noise ratio of the neural representation [22], [23].

Effect (C). The perceived masker intensities might be factored into the decision, in the sense that the decision variable depends not only on the representations of target intensity, but also on the representations of masker intensity.

Effects A and B correspond to a masker-induced change in the representation of target intensity. Notably, if effect A caused the same change in the internal representation for both target tones in a two-interval (2I) task, then the performance in the discrimination task would remain unaltered. In contrast, effect B would invariably cause impairment in intensity resolution. Effect C differs from effects A and B in that the representations of *masker* intensity are included in the decision variable. This would mean that the decision is not exclusively based on a comparison of the two representations of target intensity in a 2I task, but that the perceived masker intensities are factored into the decision. Effect C would result in impaired performance even in the absence of effects A and B, because the maskers provide no information concerning the correct response but the variance of the masker representations will be added to the decision variable. This variance encompasses internal noise as well as "external noise" due to for example trial-by-trial variation in masker level as in the present experiment. Effects A, B, and C might of course also operate in parallel.

A two-interval discrimination task represents a situation with multiple observations. Therefore, the potential masker effects A, B, and C can be related to a simple observer model based on a signal-detection theory framework for multiple observation tasks [14]. The model assumes two processing stages. In the first stage ("sensory processing"), each tone (masker and target in first interval, masker and target in second interval) is processed by the auditory system, resulting in a separate representation of the sound intensity of each of the four tones presented per trial. These representations are stored in a memory system. The second stage (decision stage) then combines the level representations and selects a response according to some decision rule. For reasons of simplicity, we conceive the combination of information to be lossless, i.e., in the signal detection theory tradition there is no decision noise. We further assume that the decision variable (i.e., value on the internal continuum) is a weighted sum of the representations of the four tone levels presented on a given trial, with unobservable decision weights. Now, the information about target intensity available at the decision stage might be impaired because the maskers reduce the precision of the information about the intensity of the targets, which is modeled as an increase in *internal noise* (effect B). The maskers could also distort the representation of target intensity in the first stage (effect A), that is, before information integration. Alternatively, it might be the case that a precise representation of target intensity is available at the decision stage (i.e., effects A and B are absent), but that this information is not used in an optimal fashion [14]. In particular, the decision variable might be influenced by the representations of the masker intensities (effect C), which provide no information concerning the correct response and should therefore not be included in the decision variable. A systematic influence of the masker levels on the decision could either be due to a direct influence of the maskers on the representations of target intensity in the first stage (effect A), or to the inclusion of masker intensity information in the decision variable in stage 2 (effect C). The latter effect corresponds to non-zero decision weights assigned to the masker intensity representations. On the behavioral level, both effects would result in a systematic relation between the (randomly varying) masker levels and the response, as observed in previous studies from our lab [24], [25].

**Figure 1:**
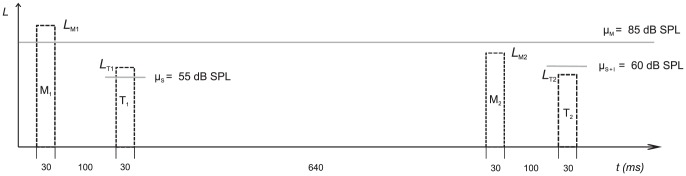
Stimuli. Schematic depiction of a trial. In a two-interval auditory intensity discrimination task, the two target tones (*T_1_* and *T_2_*) were combined with two forward maskers (*M_1_* and *M_2_*). All tone levels were independently and randomly perturbed. The gray horizontal lines show the mean levels for maskers (µM), standard (µ_S_), and standard-plus-increment (µ_S+I_). The level increment (µ_S+I_−µ_S_ = 5 dB in this example trial) was individually selected for percent correct in the range from 70% to 85%. It was presented in either the first or the second interval with equal probability. The dotted rectangles depict the randomly selected tone levels in the example trial (*L*_M1_ to *L*_T2_).

In the present experiment, methods of "molecular psychophysics" [18], also known as perceptual weight analysis or behavioral reverse correlation [26], [27], [28], [29], provided a rich set of behavioral measures that made it possible to test which of the three effects play a role for intensity discrimination under forward masking. In a two-interval intensity discrimination task (see **Figure 1**), we imposed random trial-by-trial variation on the levels of the maskers presented in the first interval (*L*_M1_) and in the second observation interval (*L*_M2_). Now assume that the masker presented in interval 1 shifted the representation of the target intensity in interval 1 towards lower values, for instance due to neural response suppression, corresponding to effect A. Also assume that the amount of response suppression increases with the sound pressure level of the masker, as has been reported for auditory nerve neurons [19], [30], [31]. As a consequence, the probability of responding that the louder target tone had been presented in interval 1 should be *negatively related* to the (randomly varying) level of the masker in interval 1 (*L*_M1_). The same negative relation between *L*_M1_ and the probability of selecting the first interval would result if the representation of masker intensity entered the decision variable with a negative decision weight (effect C). In contrast, if the masker caused a shift of the representation of target intensity towards higher values [13], [20], or if the representation of masker intensity entered the decision variable with a positive decision weight, then the probability of selecting the first interval would be *positively related* to *L*_M1_. Thus, by quantifying the influence of the variation in masker level on the decision, it is possible to decide which of the potential effects of the maskers (e.g., response suppression versus enhancement) are compatible with the observed responses. The methods used in the present experiment quantify this relation in terms of *perceptual weights* (formally defined by the *w*-terms in Eq. (1) in section *Results*), which should be distinguished from the unobservable decision weights the listener applies when combining the representations of the masker and target intensities into the decision variable, according to the observer model introduced above.

The experimental approach also provided an estimate of internal noise, this estimate being independent of the estimates of the perceptual weights [28]. We compared the observed amount of internal noise effective in the forward masking conditions to the level of internal noise that would have resulted if the maskers had influenced the performance in terms of effects A and C, but had not caused an increase in the internal noise effective for the targets (i.e., no effect B present). To this end, we included conditions presenting the targets in quiet (without maskers), and used the estimates of the internal noise effective in quiet in the analysis.

To summarize, the perceptual weights measuring the relation between the presented and randomly varying target and masker levels and the response in the intensity discrimination task provide information concerning whether the maskers caused systematic shifts of the decision variable, either by directly altering the representations of target intensity (effect A), or by an inclusion of the representations of masker intensity in the decision variable (effect C). The sign of the perceptual weights also tells us whether the influence of masker intensity is compatible with response suppression or rather with response enhancement. The estimates of internal noise allow answering the question whether the maskers caused an increase in the variance of the target representations (effect B). Based on previous results suggesting an important role of attentional mechanisms [4], [5], [6], [24], [25], we expected a systematic contribution of masker intensity to the decision (effects A or C, evident in non-zero perceptual weights assigned to the maskers), but only a weak increase in the internal noise effective for the targets. We also expected the perceptual weights assigned to the maskers to be correlated with the masker-induced impairment in performance (i.e., the DL elevation).

As evident from the preceding discussion, the molecular psychophysics approach is capable of differentiating effects A or C from effect B, but cannot be used to decide between effects A and C because both effects may result in the same pattern of perceptual weights. While effect A might be caused at the earliest auditory processing stages (e.g., by response suppression in the auditory nerve), the information integration represented by effect C is likely to be located at higher stages in the auditory pathway. In order to gain information about the extent to which the effects of the maskers involve peripheral and higher stages, we varied the lateralization of the maskers relative to the targets via the interaural time difference (ITD). We expected the difference in lateralization to promote object separation between masker and target, which should facilitate selective attention to the target [16] and therefore result in smaller decision weights assigned to the maskers. This would correspond to a reduced influence of effect C, compatible with a previous study from our lab that found smaller DL elevations with contralateral than with ipsilateral maskers [5]. Notably, as we varied only the masker ITD, the waveform delivered to each of the two ears (i.e., the monaural channels) was identical in the conditions with ipsilateral and contralateral masker. This ensured that the representation of masker and target in the auditory nerve did not differ between the two masker lateralizations. Thus, any observed differences in perceptual weights, estimated internal noise or intensity DLs between the ipsilaterally and contralaterally presented maskers can be attributed to mechanisms located in the superior olivary complex (SOC; first binaural interaction in the ascending auditory pathway) or at higher stages.

To summarize, the aim of the present study was to identify effects of forward masking on the behavioral decisions in an auditory intensity discrimination task. The results show that the effects of forward masking on intensity discrimination are largely due to an inclusion of masker information in the decision variable, rather than to masker-induced increases in internal noise. The influence of the masker intensities on the decision, measured by the ratio between the perceptual weights assigned to maskers and targets, explained a reasonably high proportion of variance of the impairment in performance caused by the maskers (*R* ^2^ = .72). We propose that our results are compatible with object-based attention.

## Methods

The listeners were tested in a two-interval, two-alternative forced-choice (2I, 2AFC) intensity discrimination procedure. Pure-tone standards (500 Hz) with a sound pressure level of 30, 55 and 85 dB SPL were presented in quiet, and combined with an 85 dB SPL forward masker (500 Hz). All tones were presented binaurally. The lateralization of the maskers relative to the targets was varied via the inter-aural time difference (ITD), so that the maskers were either perceived on the same side of the head as the targets (ipsilateral maskers), or on the other side (contralateral maskers). To estimate the contribution of the maskers and targets to the decision, the two masker levels as well as the two target levels presented on each trial were randomly and independently perturbed, as explained below.

### Participants

Seven students at the Johannes Gutenberg – Universität Mainz participated in the experiment voluntarily (5 female, 2 male; age range 20–36 years). They either received partial course credit or were paid for their participation. All listeners reported normal hearing. Detection thresholds measured by Békésy tracking [32], [33] with pulsed 270-ms tones including 10-ms cos^2^ on- and off-ramps were better than 20 dB HL between 125 Hz and 4 kHz for both ears. Three listeners had previous experience with comparable psychoacoustic tasks.

### Ethics statement

The experiment was conducted according to the principles expressed in the Declaration of Helsinki. All listeners participated voluntarily after providing informed written consent, after the topic of the study and potential risks had been explained to them. They were uninformed about the experimental hypotheses. The study was approved by the ethical review board of the Department of Psychology at the Johannes Gutenberg-Universität Mainz.

### Stimuli and apparatus

The standard and the masker were 500 Hz pure tones with a steady-state duration of 20 ms, gated on and off with 5-ms cosine-squared ramps. Each sinusoid started at zero phase. On each trial, there were two observation intervals. Except in no-increment trials (see below), an increment— that is, a pure tone of the same frequency, duration, and temporal envelope—was added in-phase to the standard in one of the observation intervals (selected with an equal *a priori* probability). In the forward masking conditions, a masker was presented in both intervals. The silent interval between masker offset and target onset was 100 ms. In the in-quiet condition, the maskers were omitted. The temporal interval between the onsets of the two target tones (standard and standard-plus-increment) was 800 ms. On each trial, the sound pressure levels of the masker presented in interval 1 and of the masker presented in interval 2 were sampled independently from the same normal distribution. The mean of the distribution was µM = 85 dB SPL, its standard deviation was *SD_M_* = 2.5 dB. The masker levels were limited to a range of µM ±2.5 *SD_M_* to avoid unduly high sound pressure levels. The levels of the targets presented in interval 1 and interval 2 were also sampled independently from a normal distribution on each trial. The mean of the distribution was varied (µ_S_ = 30, 55 or 85 dB SPL). In the first part of the experiment, intensity DLs were measured with an adaptive procedure and the target level was fixed. Put differently, the standard deviation of the target level was *SD_S_* = 0 dB. In the main part of the experiment used for the estimation of perceptual weights, the target tone levels were randomly perturbed in the same way as the masker levels. Here, the standard deviation of the normal distributions from which the target levels were sampled was *SD_S_* = 2.5 dB. The same range restriction as for the maskers was applied. The targets were presented binaurally with an interaural time difference (ITD) of +500 µs (i.e., the waveform presented to the right ear started 500 µs earlier than the signal to the left ear). For the 500 Hz tones this corresponds to an interaural phase difference of 90°. The targets were perceived as lateralized to the right side of the head. The maskers were presented either with the same ITD as the targets (ipsilateral), or with an ITD of −500 µs (contralateral) and were therefore lateralized either to the same side as the targets, or to the opposite side (see section *Perceived lateralization* below).

A trial started with a visual attention signal. The targets (standard and standard-plus-increment) were also marked by visual signals. The inter-trial interval was 2000 ms, with the restriction that the next trial never started before the response and the feedback to the preceding trial had been given. The stimuli were generated digitally, played back via two channels of an RME ADI/S digital/analog converter (*f*_s_ = 44.1 kHz, 24-bit resolution), attenuated by a TDT PA5 programmable attenuator, buffered by a TDT HB7 headphone buffer, and presented both ears via Sennheiser HDA 200 circumaural headphones calibrated according to IEC 318 [34]. The experiment was conducted in a double-walled IAC sound-insulated chamber. Listeners were tested individually.

### Procedure

#### Adaptive measurement of intensity-difference limens

In the first phase of the experiment, intensity DLs were measured using a 2I, 2AFC adaptive procedure with a 2-down, 1-up tracking rule [35]. A level increment was added to the standard in one of the two randomly selected observation intervals. No random level perturbation was applied to the targets, but the masker levels were randomly perturbed as described above. Listeners were instructed to ignore the maskers. Visual trial-by-trial feedback was provided. The initial level of the in-phase intensity increment was 10 dB, in terms of 10 log_10_(Δ*I*/*I*). The step size was 5 dB until the fourth reversal, and 2 dB for the remaining eight reversals. For each track, the arithmetic mean of 10 log_10_(Δ*I*/*I*) at the eight final reversals was taken as the DL estimate, corresponding to 70.7% correct. A track was discarded if the *SD* of 10 log_10_(Δ*I*/*I*) at the eight final reversals was greater than 6 dB. Five blocks were presented for each Mean Standard Level × Masker Lateralization combination, in separate sessions. For a given listener and condition, DL estimates more than 1.5 times the interquartile range lower than the first or higher than the third quartile were classified as outliers [36], resulting in the exclusion of at most two data points per listener and condition. The order of conditions was randomized in each session. The DLs measured in this task (sessions 4–8) were used to select individual intensity increments for the main task in which perceptual weights were estimated. Only the data from the main task (see next section) were used to test the hypotheses.

#### Estimation of perceptual weights and internal noise: Intensity discrimination with random level perturbations

In a 2I, 2AFC procedure, a level increment Δ*L* was added to the standard in one of the two observation intervals (selected randomly). The temporal structure of a trial is depicted in **Figure 1**. Listeners selected the interval containing the louder target. They were instructed to ignore the maskers. Based on the DLs obtained in the adaptive procedure described above, an increment Δ*L* was selected individually for each combination of Mean Standard Level (30, 55 and 85 dB SPL) and Masker Lateralization (ipsilateral, contralateral, in quiet) that would correspond to percent correct in the range from 70% to 85%. The level increment was fixed within each block. Across listeners and conditions, the level increments expressed in terms of 10 log_10_(Δ*I*/*I* +1) ranged from 0.68 dB to 23.2 dB. The three mean standard levels were presented in quiet and were combined with one masker level (85 dB SPL). The masker ITD was varied so that the maskers were either perceived on the same side of the head as the targets (ipsilateral) or on the other side of the head (contralateral). Only one Mean Standard Level × Masker Lateralization combination was presented in each block. Each block comprised 35 trials with the level increment presented in the first interval, 35 trials with the increment presented in the second interval, and 35 trials without an increment. The latter condition was included to gain insight into the question of whether the size of the level increment might affect the perceptual weights, although we did not expect this to be the case because the weights assigned to the maskers were rather similar across increment position in a previous study from our lab using similar stimuli [24]. Note that due to the random level perturbation, the target levels presented in the two observation intervals also differed in tracks without increment. As a result, it was possible to provide visual trial-by-trial feedback concerning the correctness of the response on all trials, based on the actual target levels presented on a given trial. At least six blocks of 105 trials each were obtained for each Mean Standard Level × Masker Lateralization combination, in separate sessions (9–17). The order of conditions was randomized in each session. The data from this task were used to obtain maximum-likelihood estimates of the parameters of the observer model described in section *Observer model: Estimates of perceptual weights and internal noise* below.

#### Measurement of detection thresholds

Detection thresholds were obtained for 500 Hz tones presented binaurally with an ITD of +500 µs. The tones had a steady-state duration of 20 ms, and were gated on and off with 5-ms cos^2^-ramps. In a 2I, 2AFC task, the signal was presented in quiet and with a forward masker (500 Hz, 20 ms steady-state duration, 5 ms cos^2^-ramps), which was either presented with the same ITD as the signal (+500 µs), or with an ITD of −500 µs. In the forward masking conditions, a masker was presented in both intervals. The silent interval between masker offset and signal onset was 100 ms. As described above, on each trial the masker level in the first and in the second interval was sampled randomly and independently from a normal distribution with mean µM = 85 dB SPL and *SD_M_* = 2.5 dB. No random perturbation was applied to the signal level. An adaptive procedure with a two-down, one-up rule was used [35]. In one interval (selected randomly), the signal was presented, while no signal was presented in the other interval.

Initially, the signal level was 30 dB SPL. The step size was 8 dB until the fourth reversal, and 2 dB for the remaining eight reversals. Visual trial-by-trial feedback was provided. The threshold level was computed as the arithmetic mean of the signal levels at the final eight reversals. A track was discarded if the standard deviation of the latter signal levels was greater than 6 dB. For each condition, five adaptive blocks were obtained in sessions 4–8.

#### Measurement of perceived lateralization

As a manipulation check, the perceived lateralization of the tones was measured in session 3. First, for a 500 Hz, 55 dB SPL tone with an ITD of 0 µs, the ILD corresponding to lateralization exactly in the center of the head was determined via an adaptive procedure. On each trial, the listener responded whether he or she heard the tone to the left or to the right of the center of the head. The ILD was adjusted by a simple up-down rule [35]. Three such blocks were run, and the individual average ILD from these blocks was used for the main experiment (discrimination task and detection task).

The individual ILD was also used in the remaining blocks in session 3, in which the listener rated the lateralization of the tones on a horizontally oriented 41-point scale ranging from −20 (left ear) to +20 (right ear) (cf. [37]). On each trial, tones corresponding to three trials in the intensity discrimination task were presented. For example, three pairs of 55 dB SPL tones corresponded to three trials of the discrimination task for a 55 dB SPL standard in quiet. Ratings of the perceived lateralization were obtained for 30, 55, and 85 dB SPL tones in quiet, presented with ITDs of −500, 0, and +500 µs. As a control condition, the tones were additionally presented monaurally, either to the left or to the right ear. Three ratings were obtained per condition, in random order.

Next, the six different masking conditions from the intensity discrimination task were presented. On each trial, the listener heard three trials as in the intensity discrimination task, and first rated the perceived lateralization of the target tones, and then the perceived lateralization of the maskers. Three ratings of target and masker lateralization were obtained per condition, in random order.

#### Sessions

Each listener participated in a total of 17 experimental sessions, each with a duration of approximately 55 minutes. In session 1–3, practice blocks for all conditions in the intensity discrimination task and the detection task were run. Additionally, audiometric thresholds were measured in session 1. In session 3, an individual ILD was determined and the participants provided ratings of the lateralization of the tones in the different ITD conditions.

In sessions 4 to 8, intensity DLs were measured with an adaptive procedure and without random perturbation of the target levels (in each session: one block per condition, random order). These DLs were used to select individual intensity increments for the main task. Additionally, detection thresholds in quiet and under forward masking were obtained (in each session: one block per condition, random order).

In sessions 9 to 17, the intensity discrimination task with random perturbation of the target levels and a fixed intensity increment was run (six randomly selected conditions per session with the restriction that no condition was presented more than once per session, in random order).

## Results

### Intensity-difference limens

For the discrimination task with fixed level increments and random level perturbation, DLs (10 log_10_ [Δ*I/I* +1]) were estimated for each block by fitting a cumulative-normal psychometric function (PMF) relating the level difference between the target in interval 2 and the target in interval 1 to the observed probability of a "Louder tone in interval 2" response. We used a maximum-likelihood approach for fitting the PMF. Each fit provided an estimate of the mean of the cumulative-normal distribution function (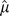; representing the point of subjective equality), and an estimate of its SD (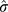), representing the spread of the PMF. We defined the DL as half the difference between the 75%- and the 25%-point on the PMF, which for a cumulative-normal PMF is given by DL = 0.67449 • 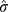. The same criterion for outlier detection as above was applied, resulting in the exclusion of at most two blocks per listener and condition. Time permitting, additional blocks were obtained in conditions where outliers were detected.

**Figure 2:**
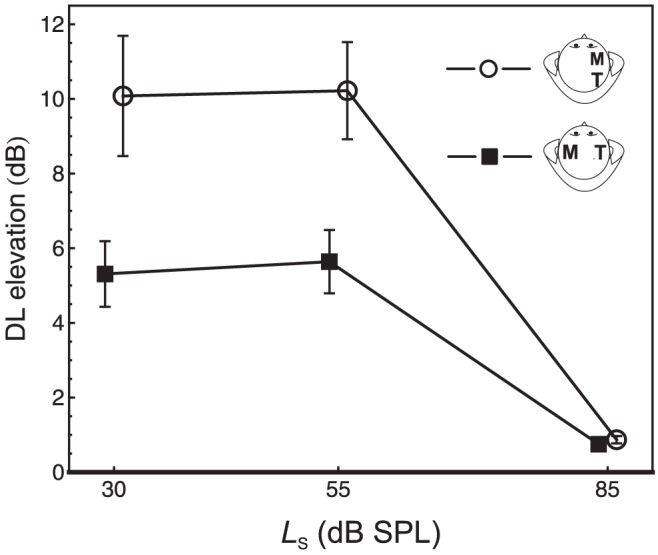
DL elevation. Average masker-induced DL elevation, representing the difference between the DL (10 log_10_[Δ*I/I* +1]) under forward masking and in quiet. Circles: maskers lateralized to the same side of the head as the standard. Squares: maskers lateralized to the contralateral side of the head as the standard. Error bars: 95%-CIs.

The data were analyzed in terms of the DL elevation, which denotes the difference between the DL under masking and the DL in quiet. The average DL elevations are displayed in **Figure 2**. A two-factorial repeated-measures analysis of variance (rmANOVA) with Huynh-Feldt correction to the degrees of freedom (cf. [25]) showed a significant effect of masker lateralization, *F*(1, 6) = 15.17, *p* = .008, η^2^_p_ = .72. As seen in **Figure 2**, the DL elevation was smaller for the contralateral maskers compared to the ipsilateral maskers, except for the case of target and masker with the same mean sound pressure level (85 dB SPL), confirming the expected smaller effect of the contralateral masker. For the two lower standard levels, the DL elevation was on average 4.67 dB (*SD* = 3.24) smaller with contralateral compared to ipsilateral maskers. This difference was significant, *t*(6) = 3.82, *p* = .009, and represents a strong effect, Cohen's [38] *d*_z_ = 1.44. The effect size was somewhat stronger than in our previous study presenting comparable masker and target levels [5]. The effect of standard level was also significant, *F*(2, 12) = 7.46, *p* = .008, η^2^_p_ = .55, Huynh-Feldt correction factor 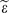 = 1.00. The DL elevation was higher at the two lower standard levels, which is the expected pattern [7]. On average, a midlevel hump (i.e., smaller DL elevation at *L*_S_ = 30 dB SPL than at *L*_S_ = 55 dB SPL) was not observed. In the individual data, midlevel humps were present for three listeners, while for the remaining listeners the DL elevation was either very similar for the 30 and 55 dB SPL standards, or highest for the 30 dB SPL standard. These individual differences are compatible with previous data (cf. [3]). The masker lateralization × standard level interaction was significant, *F*(2, 12) = 4.14, *p* = .043, 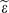 = 1.00, η^2^_p_ = .41. Three post-hoc paired-samples *t*-tests were computed to analyze the difference between the DL elevations for ipsilateral and contralateral maskers for each standard level. This difference was significant at the two lower standard levels (*p*<.05, two-tailed), but not at the 85 dB SPL standard level.

### Observer model: Estimates of perceptual weights and internal noise

We used a maximum-likelihood approach to estimate the perceptual weights and the amount of internal noise from the trial-by-trial data. Compatible with the observer model described in the introduction, we assumed the decision variable to be given by
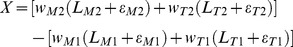
(1)where *L*_M1_ is the (randomly varying) sound pressure level of the masker presented in the first observation interval on a given trial (see **Figure 1**), *L*_T1_ is the sound pressure level of the target in the first interval (including the level increment when the latter is presented in the first interval), *w*_M1_ and *w*_T1_ are the perceptual weights assigned to the masker and the target in the first interval, respectively, and ε_M1_ and ε_T1_ are random variables representing the internal noise effective for masker and target, respectively, in interval 1. The internal noise components ε_M1_ and ε_T1_ were assumed to be independent and normally distributed with mean 0 and standard deviation σ_IM1_ and σ_IT1_, respectively. Thus, the terms in the second bracket in Eq. (1) represent a weighted sum of the masker and target levels presented in interval 1, in the presence of additive internal noise. A systematic influence of the representation of masker level on the decision variable due to effects A or C would be evident in a non-zero estimate of the perceptual weight *w*_M1_. Analogously, the terms in the first bracket in Eq. (1) represent the internal representation of the second observation interval. The listener was assumed to respond that the louder target had been presented in the second interval if *X* > *c*, where *c* is a constant representing the decision criterion.

Given these assumptions, for the intensity discrimination task under forward masking the mean and standard deviation of the cumulative-normal psychometric function relating the decision variable *X* and the probability of a "Louder target in interval 2" response are µ_FM_ = (*w*_M2_ *L*_M2_ + *w*_T2_ *L*_T2_) − (*w*_M1_ *L*_M1_ + *w*_T1_ *L*_T1_) and 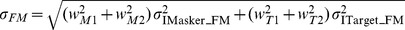, respectively, assuming that the internal noise does not differ between the two maskers or the two targets (i.e., σ_IM1_ = σ_IM2_ = σ_IMasker_FM_; σ_IT1_ = σ_IT2_ = σ_ITarget_FM_).

The probability of selecting the second interval is
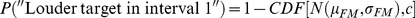
(2)where CDF[*N*(µ,σ),*c*] is the cumulative distribution function of a normal distribution with mean µ and standard deviation σ, evaluated at the point *c*. The probability of selecting the first interval is 1 − *P* ("Louder target in interval 2").

This observer model was used to obtain maximum likelihood estimates of the perceptual weights, and of the standard deviation of the decision variable under forward masking (σ_FM_). For each trial, given the four presented levels (*L*_M1_, *L*_M2_, *L*_T1_, and *L*_T2_), the likelihood of the observed response is given by Eq. (2). Assuming independence between trials, the total likelihood is the product of the likelihoods of the individual trials. We minimized the negative log likelihood numerically using the Mathematica 9.0 function *NMinimize[]*. The weights can only be estimated up to a multiplicative constant [28], which presents no problem because we were only interested in the relative weights assigned to the four tone levels. Therefore, without loss of generality, we set *w*_T2_ = 1 when fitting the model, which reduced the number of free parameters by one. As demonstrated formally by Berg [28], the estimated relative weights are independent of additive internal noise. This is obvious if one considers that in Eq. (2) the internal noise variances only appear in a common term representing the standard deviation of the normally distributed decision variable. Therefore, increasing for example σ_IT1_ will flatten out all "conditional on a single stimulus" (COSS) [28] psychometric functions that describe the relation between for example *L*_M1_ and *P*("Louder target in interval 2"), regardless of the level of the other tones (*L*_T1_, *L*_M2_, and *L*_T2_). However, the increase in σ_IT1_ will not affect the *ratios* between the estimated weights, e.g., *w*_T1_/*w*_M1_. Thus, the estimates of the relative perceptual weights on the one hand and of the internal noise SD on the other hand can be used to unequivocally and quantitatively answer the question to which extent the decision was systematically influenced by the masker intensities (effects A and C), and to which extent the maskers caused an increase in internal noise (effect B).

Separate model fits were obtained for each combination of listener, standard level, masker lateralization, and increment position (increment presented in the first interval, increment presented in the second interval, no increment). For 19 of the 189 cases, either the model did not converge, or the weight estimates were very imprecise (standard error >1.5). We excluded these cases and analyzed the weights and the internal noise SD with a general linear mixed model based on a maximum-likelihood approach (SAS PROC MIXED). This analysis can be used in the case of missing data [39]. We used the Kenward and Roger [40] solution for the degrees of freedom, which was demonstrated to be superior to alternative methods of computing the degrees of freedom [41], [42], [43]. We fitted a covariance matrix of type "heterogeneous compound symmetry" (CSH [44]) because the model did not converge with an "unstructured" (UN) matrix placing no constraints on the variance-covariance matrix. For the sample size of our study (*N* = 7), the Type I error rate when fitting a CSH covariance structure in the case of normal data can be considered as robust [25]. In the analysis, the within-subjects factors were standard level, masker lateralization, and increment position. Neither for the ratio between the masker and the target weights (see below), nor for the internal noise SD was the effect of increment position or any interaction effect involving increment position significant. Therefore, we used a meta-analytic approach to pool the estimates of the perceptual weights and of σ_FM_ across the three increment positions for further analyses. The combined estimate was computed as the weighted average of the three separate estimates, with weights inversely proportional to the variance of each estimate [45]. The variance of this combined estimate is the inverse of the sum of the inverse variances [45]. This step was conducted for each combination of listener, standard level, and masker lateralization.

#### Perceptual weights

**Figure 3:**
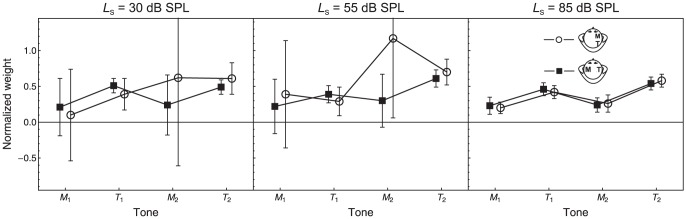
Perceptual weights. Average normalized perceptual weights for the four tones in the different experimental conditions presenting forward maskers. Circles: Ipsilateral maskers. Squares: Contralateral maskers. Error bars: 95% CIs.

To facilitate the comparison of perceptual weights across listeners, the perceptual weights were normalized so that the sum of the absolute values of the four perceptual weights was 1.0 for each combination of listener and condition [27]. **Figure 3** shows the average normalized perceptual weights for the four tones in the conditions presenting a contralateral or an ipsilateral forward masker. As evident in the 95% confidence intervals, across listeners the weights assigned to the target tones (*T*_1_: target in interval 1, *T*_2_: target in interval 2) were significantly higher than 0 in all conditions. Thus, the listeners used the task-relevant information about target intensity. However, the average weights assigned to the maskers (*M*_1_ and *M*_2_) were also higher than 0 in all conditions, showing that the decision was systematically influenced by the task-irrelevant masker information. This is evidence for the presence of effects A or C. The large confidence intervals indicate pronounced inter-individual differences in the masker weights, compatible with previous results [24]. At the 55 dB SPL standard level, 13 of 14 (listener × masker lateralization) individual weights for masker 2 were positive, and 8 of these weights were significantly different from 0 (*p*<.05, two-tailed). Nine weights for masker 1 were positive and five were negative. Three and one, respectively, of the seven masker 1 weights were significantly different from zero for the ipsilateral and contralateral masker. At the 30 dB SPL standard level, six weights for masker 1 were positive and eight were negative. Four negative weights and three positive weights differed significantly from 0. For masker 2, eight weights were positive (four significant) and 6 were negative (one significant). At the 85 dB SPL standard level, 13 of the 14 weights for masker 1 and 12 of the 14 weights for masker 2 were significantly greater than 0. However, the average masker weights were rather small at this standard level. Thus, the majority of masker weights were positive, although some negative weights were observed, especially for masker 1 and at the lowest standard level. Due to the large inter-individual variation, the mean masker weights did not differ significantly from zero.

**Figure 4:**
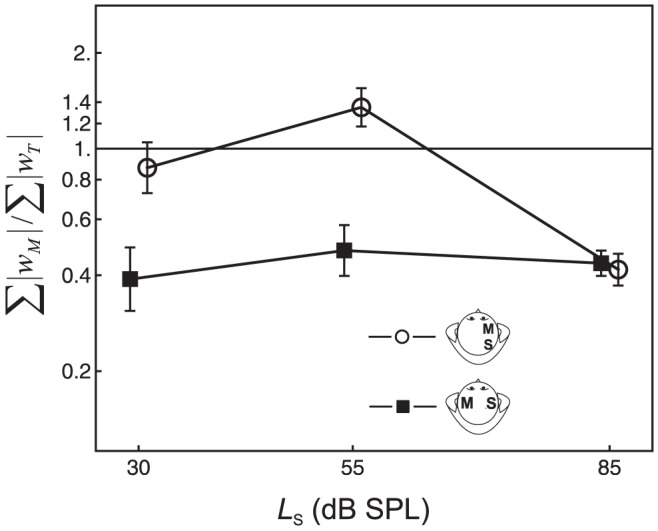
Masker-target weight ratio. Average values of the masker-target weight ratio (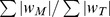), as a function of standard level. Circles: Ipsilateral maskers. Squares: Contralateral maskers. Error bars: 95% CIs.

To test the hypothesis that the strength of the influence of masker information on the decision depends on the masker lateralization and on the standard level, we analyzed the ratio 
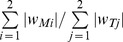
, that is, the sum of the absolute values of the two masker weights divided by the sum of the absolute values of the two target weights. If the response were completely unrelated to the masker levels, then this ratio would be 0. **Figure 4** shows the average values of the masker-target weight ratio 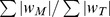. For the repeated-measures ANOVA, a log-transform was applied to the masker-target weight ratio in order to make the distribution more symmetric [25]. The within-subject factors were masker lateralization and standard level. As expected, there was a significant and strong effect of masker lateralization, *F*(1, 8) = 8.26, *p* = .026, η^2^_p_ = .58, *d*_z_ = 1.09. At the two lower standard levels, the weights assigned to the maskers were considerably smaller than the weights assigned to the targets if the masker was perceived contralaterally to the target (boxes in **Figure 4**). However, the maskers received approximately the same weight as the targets if the masker was perceived ipsilaterally (circles in **Figure 4**). This pattern of results indicates that effects A and/or C were reduced by the spatial separation between masker and target, compatible with our hypothesis that the lateralization difference between masker and target facilitates selective attention to the target. At the 85 dB SPL standard level, the weights assigned to the maskers were smaller than the target weights assigned to the targets for both masker lateralizations. As a reminder, the DL elevation caused by the maskers was very small at this standard level (see **Figure 2**). The standard level × masker lateralization interaction was significant, *F*(2, 12) = 11.6, *p* = .005, 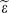 = .70, η^2^_p_ = .66. The effect of standard level was not significant, *F*(2, 12) = 2.41, *p* = .158.

#### Relation between the masker-target weight ratio and the DL elevation

**Figure 5:**
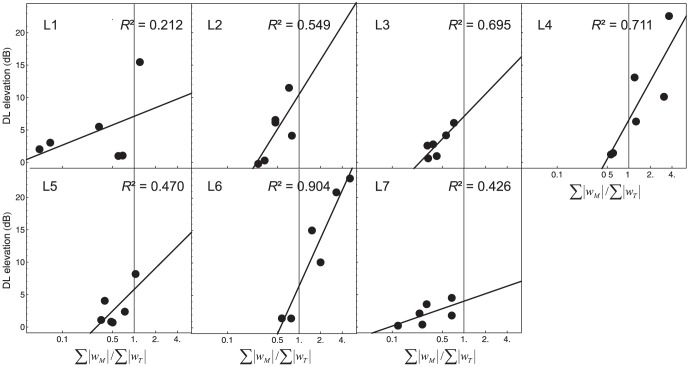
Masker-target weight ratio as a predictor of the DL elevation. Best-fitting individual linear regression lines for the relation between the masker-target weight ratio and the DL elevation in the six conditions (standard level × masker lateralization). Each panel represents one listener.

According to our hypotheses, the DL elevation caused by the forward maskers can for a large part be attributed to a systematic influence of the masker intensities on the decision variable (effects A or C). Because the masker-target weight ratio 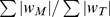 is a quantitative measure of this influence, it follows from our hypothesis that the DL elevation should be strong in cases where 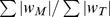 is high. To test this prediction, we computed the linear regression between 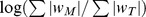 and the DL elevation, separately for each listener. The best-fitting individual linear regression lines through the six (standard level × masker lateralization) data points are shown in **Figure 5** together with *R* ^2^, the proportion of variance accounted for by the linear regression models. Except for listener L1, the linear regression accounted for a moderate to high proportion of the variance. To summarize this relation across listeners, we analyzed the data using a random effects model with random intercept and slope (cf. [46]), taking into account the repeated-measures structure of the data. The variance-covariance matrix was specified as being of type "unstructured" (UN; [44]), and the degrees of freedom were computed according to the method by Kenward and Roger [40]. This analysis showed a significant positive linear relation between the DL elevation and the log masker-target weight ratio, *F*(1, 6.3) = 16.36, *p* = .006. The coefficient of determination computed according to Edwards et al. [47] was *R* ^2^_β_ = .72. This analysis shows that the influence of the masker intensities on the decision variable (effects A or C), quantified by the masker-target weight ratio, accounted for a high proportion of the variance in DL elevation. This result is compatible with our hypotheses.

#### Internal noise

The above analyses of the perceptual weights provided evidence for an inclusion of masker information in the decision variable due to effects A or C, showed that this influence depends on the standard level and on the masker lateralization, and demonstrated that higher weights assigned to the maskers correspond to stronger DL elevations. The remaining question is: Did the maskers additionally cause an impairment of the representations of target intensity available at the decision stage, that is, an increase in the internal noise effective for the targets? In other words, is there evidence for a presence of effect B? This question can again be answered using the observer model specified in Eq. (2), and by relating the internal noise effective in quiet to the observed variance of the decision variable under forward masking.

The internal noise effective in the in-quiet condition can be estimated from the trial-by-trial data obtained in quiet by assuming σ_IT1_ = σ_IT2_ = σ_ITarget_quiet_, and setting *w*_M1_ = *w*_M2_ = 0 in Eq. (1). For the forward-masking condition, it is unfortunately not possible to obtain separate estimates of σ_IMasker_FM_ and σ_ITarget_FM_, because in Eq. (2) these parameters appear only in a common term. Yet, we can use the ML-estimate of σ_FM_ obtained by fitting the model (Eq. (2)) for a test of whether the internal noise was increased in the forward masking condition, relative to the in-quiet condition. To this end, we compared the estimate of σ_FM_ to the quantity 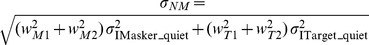, where *w*_M1_, *w*_M2_, *w*_S1_ and *w*_S2_ are the perceptual weights estimated in the forward-masking condition, and 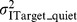 and 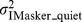 are the internal noise variances estimated for the corresponding tone levels (30, 55 and 85 dB SPL) in the in-quiet conditions. We used the estimated internal noise variance for the 85 dB SPL targets presented in quiet as the estimate of 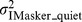 because the maskers and targets had the same frequency and duration, and because we did not expect the ITD to have an effect on the internal noise variance. Now, σ_NM_ is the SD of the decision variable under forward-masking that would result when the internal noise effective for maskers and targets were *identical* to the situation in quiet. Note that σ_NM_ includes the contribution of the masker-associated internal noise to the variance of the decision variable, which is determined by the weights assigned to the maskers. If we assume that presenting the maskers and targets together instead of in isolation can result in an increase, but not in a reduction of the internal noise effective for each of the four tones, then if σ_FM_ = σ_NM_ there was no increase in the internal noise effective for either of the four tones. In contrast, if σ_FM_>σ_NM_ then at least one of the internal noises was increased under forward masking.

For large samples (i.e., high number of trials, as in our experiment), maximum likelihood estimates are approximately normal [48], and their asymptotic variance-covariance matrix can be calculated in terms of the Fisher information, by taking the inverse of the Hessian matrix [48]. It was therefore possible to test *H*_0_: σ_FM_ = σ_NM_ on an individual basis. The ML analysis of the data from the forward-masking conditions provided estimates of the weights, their standard errors (SEs), as well as an estimate of σ_FM_ and its SE. ML analyses of the data from the in-quiet conditions provided estimates and SEs of 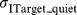 and 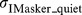. The estimate of σ_NM_ was computed as detailed above. We were unable to find an analytic solution for the standard error of σ_NM_ and therefore used a Monte Carlo approach. For each subject and combination of standard level and masker lateralization, we simulated 200,000 values of σ_NM_, with the perceptual weights (*w*_M1_, *w*_T1_, *w*_M2_, *w*_T2_), 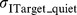, and 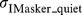 drawn from normal distributions with means and standard deviations as estimated via the ML analyses of the observed data. The mean and standard deviation of the simulated samples of σ_NM_ were taken as estimates of the population mean and standard deviation of σ_NM_. The simulated perceptual weights were multinormally distributed, with mean vector (*w*_M1_, *w*_T1_, *w*_M2_, *w*_T2_) equal to the vector of estimated weights (aggregated across increment positions using the meta-analytic approach described above), and variances as estimated by the ML analysis for a given subject and condition (again combined using the meta-analytic approach). The covariances were set to 0. The simulated "isolated" internal noises 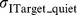 and 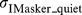 were independent and normally distributed with means and standard deviations as estimated by the ML analysis of the in-quiet conditions. For a given subject and forward-masking condition (e.g., standard level 55 dB SPL, contralateral maskers), the test statistic *z*_diff_ = 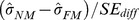, where SE_diff_ = 
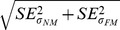
, can be referred to a standard normal distribution, providing a test of *H_0_*: σ_FM_ = σ_NM_ against *H_1_*: σ_FM_ ≠ σ_NM_.

In the in-quiet conditions, the mean estimated internal noise standard deviation was 2.61 dB (SD = 1.22 dB), 2.67 dB (SD = 1.03 dB), and 1.71 dB (SD = 0.54 dB) for the 30, 55, and 85 dB SPL standard, respectively. These values reflect the near miss to Weber's law [49]. For one listener, the estimated internal noise SDs in quiet were about twice as high as for the other listeners, at all standard levels. We have no explanation for this result. The high internal noise estimates in quiet caused σ_NM_ to be much higher than σ_FM_ for this listener, in all conditions. We excluded the listener from the analyses of the internal noise SD, because we believe his internal noise SD estimates in quiet to be erroneous.

**Figure 6:**
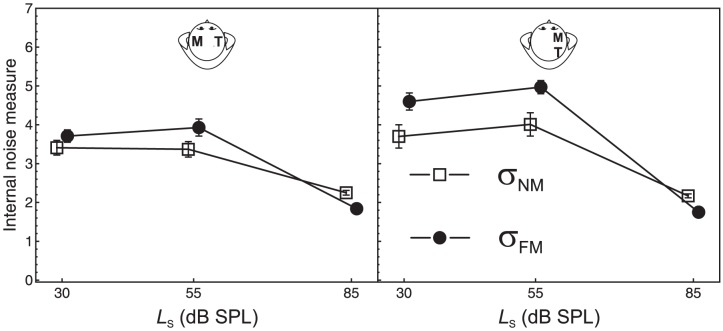
Standard deviation of the decision variable. Average estimates of the observed standard deviation of the decision variable (σ_FM_) and the SD σ_NM_ that would result when the internal noise effective for maskers and targets were *identical* to the situation in quiet. If σ_FM_>σ_NM_ then at least one of the internal noises was increased under forward masking (see the text). The SD estimates are plotted as a function of standard level and masker lateralization. Left panel: contralateral maskers. Right panel: ipsilateral maskers. Data of 6 listeners (see text). Squares: σ_NM_. Circles: σ_FM_. Error bars: 95% CIs.

The average estimates of σ_FM_ and σ_NM_ are displayed in **Figure 6**, for the remaining six listeners. Descriptively, the estimate of σ_FM_ (the observed SD of the decision variable) was slightly higher than the estimate of σ_NM_ (the SD of the decision variable corresponding to internal noise SDs for maskers and targets as low as in quiet) at the two lower standard levels. However, a repeated-measures ANOVA with the within-subjects factors SD estimate (σ_FM_, σ_NM_), standard level, and masker lateralization showed no significant effect of the type of SD estimate, nor any significant interaction involving this factor (all *p*-values >.15). Thus, the hypothesis of σ_FM_ = σ_NM_ could not be rejected. In other words, we did not find clear evidence for increased internal noise under forward-masking. Analyses of the individual data using the *z*-test explained above showed that σ_FM_ was significantly higher than σ_NM_ in only 8 of the 36 (listener × standard level × masker lateralization) cases. These results are compatible with our hypothesis that the forward maskers cause no or only a weak increase in the internal noise effective for the targets.

The rmANOVA showed a significant effect of standard level, *F*(2, 10) = 31.1, *p*<.001, 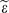 = 1.0, η^2^_p_ = .86, and a significant standard level × masker lateralization interaction, *F*(2, 10) = 6.76, *p* = .014, 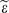 = 1.0, η^2^_p_ = .58. The effect of masker lateralization just failed to reach significance, *F*(1, 5) = 6.25, *p* = .054, η^2^_p_ = .57. These results cannot be taken as evidence for differences in internal noise between the experimental conditions, however, because the estimate of the total internal noise variance encompasses the decisions weights, which clearly differed between conditions, as demonstrated in the preceding section. Instead, the relevant measure in this case is the difference between σ_FM_ and σ_NM_, which measures the increase in internal noise in the forward-masking condition compared to the in quiet condition. An rmANOVA conducted on the difference between the two internal noise estimates (i.e., σ_FM_−σ_NM_) showed no significant effects (all *p*-values >.16), demonstrating that the standard level and the masker lateralization had no effect on internal noise, unlike for the masker-target weight ratio. This corroborates the conclusion that the variation in the masking effects (DL elevation) observed in the different experimental conditions cannot be attributed to masker-induced increases in internal noise.

Additional support of this conclusion was provided by a multiple regression analysis relating the DL elevation to the masker-target weight ratio and the increase in internal noise (σ_FM_−σ_NM_). The analysis used the same random-effects model approach as for the regression of the DL elevation on the masker-target weight ratio reported in section *Relation between the masker-target weight ratio and the DL elevation*. The regression coefficient was significant for the masker-target weight ratio (*p* = .018), but neither for the increase in internal noise (*p* = .13) nor for the interaction between the two predictors (*p* = .28).

### Detection thresholds

The same procedure for outlier detection as in the discrimination task was used. For all listeners, the detection thresholds in the forward masking conditions were below 16 dB SPL. Thus, all target tones in the experiment were presented at levels least 7.75 dB above threshold. A one-factorial rmANOVA showed no significant effect of the masking condition (in quiet, contralateral masker, ipsilateral masker), *F*(2, 12) = 2.60, *p* = .128. The average detection threshold in quiet (*M* = 9.57 dB SPL, *SD* = 2.87 dB) was only slightly lower than the detection thresholds under forward masking (ipsilateral masker: *M* = 11.28 dB SPL, SD = 2.68 dB; contralateral masker: *M* = 10.52 dB SPL, SD = 2.79 dB). The absence of a significant effect of masking condition indicates that the observed pronounced effects of masker lateralization on the DL elevation cannot be explained by differences in the detection thresholds.

## Discussion

We identified three potential effects of forward masking on performance in an auditory intensity discrimination task. The maskers might shift the mean value of the representations of target intensity (effect A), might increase the variance of the representations of target intensity (increase in internal noise; effect B), or the representations of masker intensity might be included in the decision variable (effect C). We formulated a simple observer model for a two-interval intensity discrimination task in quiet and under forward masking, based on a signal detection framework for multiple observation tasks [14]. Methods of molecular psychophysics provided independent estimates of the relative perceptual weights assigned to the four tones and of the internal noise variance [28] according to the observer model (Eq. (2)). The perceptual weight estimates quantify the relation between the response and the levels of the four tones (maskers and targets). Thus, the data provided a quantitative assessment of the influence of the masker levels on the decision, and of limitations in the precision of the representations of target intensity (i.e., increase in internal noise) introduced by the maskers.

In the experiment, we observed individual intensity DL elevations of up to 23 dB when 30 or 55 dB SPL targets were combined with 85 dB SPL forward maskers, while the same maskers caused only a small DL elevation for 85 dB SPL targets (see **Figure 2**). These results are compatible with previous data, except that on average we did not find a stronger DL elevation for standards presented at intermediate compared to low sound pressure levels [7]. We also varied the masker ITD relative to the target ITD so that the maskers were perceived either ipsilaterally or contralaterally to the target. At the two lower target levels, the DL elevation was on average 4.67 dB smaller with contralateral compared to ipsilateral maskers (*d*_z_ = 1.44), successfully replicating the effects found in a previous study presenting similar conditions [5]. Because the variation in masker ITD does not affect the representation of the stimuli in each of the two monaural channels (i.e., left and right cochlea and auditory nerve), this effect must be caused by mechanisms located in the SOC, where the first binaural interaction in the ascending auditory pathway occurs, or at higher processing stages.

The "behavioral reverse correlation" analysis of the trial-by-trial data showed a significant influence of the to-be-ignored masker levels on the responses in the discrimination task for most listeners and conditions. This result could be explained by effect A if it is additionally assumed that the masker-induced shift in the representation of target intensity depends on the (randomly varying) masker level. Alternatively, the significant perceptual weights estimated for the maskers could be caused by an inclusion of the masker representations at the decision stage where the intensity information from the four tones is integrated (effect C). Across listeners and conditions, the majority of masker weights were positive. For example, on average the probability of selecting the second target (see **Figure 1**) increased with increases in the masker level in the second interval, as evidenced by the positive perceptual weights assigned to the second masker (see **Figure 3**). This pattern is incompatible with suppression of the neural response to the target, as observed in the auditory nerve [19], [31] or in the cochlear nucleus [50]. Instead, the positive masker weights could be explained by response enhancement induced by a preceding sound, as has been observed in the auditory cortex (AC) [20], [51], [52], [53], [54], [55]. However, in studies reporting response enhancement either in neuronal recordings or in EEG or MEG responses, enhancement was mainly observed when the masker (inducer tone) and the target differed in frequency (e.g., Fig. 6 in [56]), while response suppression prevailed for on-frequency maskers like in the present experiment (see Fig. 1 in [20]). In addition, neural response enhancement in the AC appears to be caused only by forward mechanisms, not by backward mechanisms [20]. These characteristics of response enhancement in the AC are incompatible with the observation that the masker-induced DL-elevation in an intensity discrimination task is maximal for on-frequency maskers [57], and that backward maskers cause equal amounts of DL-elevation as forward maskers [4], [8]. For these reasons, the positive sign of the masker weights suggests that the influence of the masker intensities on the decision can be attributed to an inclusion of the masker representations in the decision variable (effect C) rather than to systematic shifts in the representations of target intensity (effect A).

At this point, the potential role of persistence of neuronal activation should be considered, which denotes neuronal responses continuing even after the termination of the sound stimulus. For example, a recent study reported 50 ms of persistence in the firing of auditory cortex neurons in response to pure tones with an increasing or decreasing level profile [58]. Neuronal response enhancement by a temporally adjacent stimulus is defined as a stronger response of the neuron to the target stimulus when presented together with preceding or following stimuli, compared to when the target is presented alone. For a conditioner stimulus preceding the target stimulus in time, this enhancement might be caused by persistence of activation, that is, by residual activation caused by the conditioner in the temporal window in which the neural responses to the target are measured. However, persistence can, by definition, only account for effects of forward maskers, not for effects of backward maskers. Therefore, given the very similar effects of forward and backward maskers on intensity discrimination discussed above, it is unlikely that persistence plays an important role for effect A).

In addition to the perceptual weights, the experimental method provided information about potential increases in the internal noise effective for the intensity representations of targets and maskers. To this end, we compared the observed standard deviation of the decision variable to the standard deviation that would have resulted if the internal noise SDs under forward-masking had been as low as in quiet. On average, we did not find evidence for significantly higher internal noise under forward masking, compatible with our hypothesis that effect B plays only a minor role for the masking effects on intensity discrimination performance, differently than assumed by previous models for the effects of non-simultaneous maskers on intensity resolution [7], [8], [11]. Inspection of the individual data showed, however, that for some listeners and in some conditions the maskers caused a significant increase in internal noise. It would be interesting to identify individual or stimulus-related factors influencing the presence or absence of an increase in internal noise in future experiments.

Compatible with our hypotheses, the influence of the masker intensities on the decision, measured by the ratio between the perceptual weights assigned to maskers and targets, explained a reasonably high proportion of variance for the DL elevation (*R* ^2^ = .72; also see **Figure 5**). For example, the masker-target weight ratio was significantly higher with ipsilateral than with contralateral maskers (see **Figure 4**). This result is compatible with object-based attention [16] because the lateralization difference should promote the perceptual organization of maskers and targets as separate auditory objects, making it easier to direct selective attention to the targets [4]. In contrast, the DL elevation was not correlated with increases in internal noise.

Why should it be difficult to selectively attend to the targets, given that at masker-target inter-stimulus intervals (ISIs) of 100 ms or more as in previous experiments the masker and the target presented in each observation interval are clearly perceived as two separate tones [59]? Oberfeld and Stahn [4] argued that this can be explained by the temporal structure of a two-interval intensity discrimination task. In the present experiment, the masker and the target presented in the first observation interval were separated by an ISI of 100 ms, followed by a silent interval of at least 640 ms and then the second observation interval again containing one masker and one target. Therefore, it is likely that the masker and the target in the first interval were grouped together on the basis of temporal proximity [60] and were therefore perceived as one unitary object, while the masker-target pair in the second interval was perceived as a second, separate object. In this situation, according to the concept of object-based attention, it should be difficult to selectively attend to one feature (target intensity) of the auditory object presented in interval 1 while ignoring another feature (masker intensity) of this same object. Two recent studies from our lab supported this hypothesis. In one of the experiments [4], we compared the DL elevation in the usual two-interval intensity discrimination task under forward masking to a condition where a brief sequence of three forward-maskers was presented in each observation interval. The inter-stimulus intervals between the three maskers were much smaller than the ISI between the target and its temporally most adjacent masker. Therefore, within each observation interval we expected the three forward-maskers to be grouped together on the basis of temporal proximity and thus to be processed as one auditory object, while the target should be perceived as a separate object. This should facilitate selective attention to the targets. Compatible with this hypothesis, the masker-induced DL elevation was significantly smaller in the condition presenting three maskers per interval than in the usual forward-masking condition where only one masker was presented per interval. In the latter condition, the masker-target pairs can be assumed to be perceived as one unitary object. In another experiment [4], the targets were presented in a longer regular sequence of maskers. In this condition, listeners reported to receive the maskers as one auditory stream (cf. [61], [62]) and the targets as separate events. Again, the elevation of the intensity DL caused by the maskers was significantly smaller in the streaming condition than in control conditions where the maskers and targets could be assumed to be grouped as one object. An important aspect of these experiments is that the conditions favoring the perception of maskers and targets as separate objects presented a higher number of maskers than the control conditions. Adaptation in the auditory nerve should have been stronger in the conditions presenting a higher number of maskers [63], [64], [65]. Therefore, it would be difficult to explain the observed *reduction* in DL elevation in the condition with three maskers by a reduction in peripheral adaptation.

The variation in masker lateralization by means of the interaural time difference (ITD) used in a subsequent study from our lab [5] completely avoided this potential problem by presenting conditions that differed in the expected perceptual organization of maskers and targets (one object versus two objects) but not in the representation of the stimuli in the auditory nerve. In the present experiment, we replicated the observation [5] of a significantly smaller DL elevation with contralateral than with ipsilateral maskers. In addition, we demonstrated that the effect of masker lateralization can be attributed to a reduced influence of masker information on the decision, compatible with selective attention to the targets, but not to changes in the internal noise effective for the target representations.

Although the effects of ITD variation rule out cochlear effects, channels based on spatial location can be found in the auditory pathway as low as in the SOC. Therefore, in principle the effect of masker lateralization on the masker weights might have been caused by mechanisms in the brainstem. Does this imply that object-based attention cannot be a valid explanation of the observed effects? In our view, this is not the case because the corticofugal (descending) auditory system [66] modulates response properties in the thalamus [67], [68], the midbrain [69] and even in the cochlea [70], [71]. Thus, corticofugal projections may contribute to selective attention because they can enhance neuronal responses to relevant stimuli and suppress responses to irrelevant stimuli [72], [73]. For instance, visual attention was reported to modulate the spatial tuning of auditory midbrain neurons in the barn owl [74], compatible with the strong evidence for top-down influences on visual sensory processing [75]. In fact, even if in the present experiment the weaker influence of masker information on the decision in the contralateral condition were caused by binaural processing mechanisms at the level of the brainstem, the effect can only be explained by top-down processes, because it requires task-specific knowledge that the stimuli lateralized at the right side of the head were the targets and the stimuli lateralized at the left side were the maskers.

The finding that the effects of forward maskers on intensity discrimination are largely due to an inclusion of masker information in the decision variable is compatible with results in the visual domain. In a study also applying methods of molecular psychophysics, Nandy and Tjan [76] found that effects of visual crowding in a letter identification task are mainly due to the inclusion of distractor features in the decision (source confusion or feature mislocalization), as revealed by classification images [29]. There was no evidence for masking as a cause of crowding, in the sense of the sensitivity to the target features being suppressed by the presence of the flankers. This pattern of results is similar to our observation that there was at the most a weak deterioration in the representations of target intensity (i.e., no increase of internal noise). Perceptual grouping was reported to have a strong effect on visual crowding and visual backward masking [77], [78], [79], compatible with the effect of spatial separation between maskers and targets observed in the present study. On a general level, both the auditory and the visual effects are compatible with the framework of object-based attention, but additional research is needed to evaluate alternative explanations.

It should be noted that while providing a very detailed insight into observers' performance, our methodological approach has limitations. Currently, we cannot provide a method for obtaining independent estimates of the internal noise components effective for each of the stimulus elements (i.e., ε_T1_, ε_M1_, ε_T2_, ε_M2_ in Eq. (1)). While for our data we were able to answer our research question by comparing the standard deviation of the decision variable under forward masking to the standard deviation that would have resulted if the internal noise associated with each of the four tones had been as low as in quiet, additional research would of course be desirable here.

The observer model we used and the experimental procedure we applied to estimate the model parameters are of course in no way restricted to auditory stimuli, nor to intensity discrimination tasks. The method is generally suitable for situations where decision strategies and changes in internal noise are to be differentiated. For instance, visual judgments of time-to-contact have been reported to be impaired in the presence of moving distractor objects [80], [81], with potential implications for traffic safety [82], [83], [84]. Using our experimental approach, it should be possible to answer the question of whether the additional and task-irrelevant objects reduce the precision of the representation of target time-to-contact in the visual system (i.e., increase in internal noise), or whether the performance drops because the observers' decision is influenced by time-to-contact information for the distractor objects (e.g., due to a failure of selective attention).
